# Mid-term outcome following revision surgery of clavicular non- and malunion using anatomic locking compression plate and iliac crest bone graft

**DOI:** 10.1186/s12891-017-1488-2

**Published:** 2017-03-29

**Authors:** Marc Beirer, Ingo J. Banke, Norbert Harrasser, Moritz Crönlein, Dominik Pförringer, Stefan Huber-Wagner, Peter Biberthaler, Chlodwig Kirchhoff

**Affiliations:** 1 Department of Trauma Surgery, Klinikum rechts der Isar, Technical University of Munich, Ismaningerstr. 22, Munich, 81675 Germany; 2 Clinic of Orthopedics and Sports Orthopedics, Klinikum rechts der Isar, Technical University of Munich, Ismaningerstr. 22, Munich, 81675 Germany

**Keywords:** Non-union, Malunion, Clavicle, Locking compression plate, Iliac crest bone graft, Complication

## Abstract

**Background:**

Treatment of clavicular non- and malunion is still challenging. Current surgical procedures often result in frustrating functional outcome along with high-grade subjective impairment and increased rates of revision surgery. However, the combination of biological augmentation with vital bone graft and a biomechanically sufficient fixation system seems to be a promising concept of treatment.

**Methods:**

In this retrospective study, 14 patients with a mean age of 44 years (26–67 years) suffering from non-union (*n* = 11) and/or malunion (*n* = 3) of the clavicle were enrolled. All patients were surgically treated using an anatomical precontoured locking compression plate (LCP) and autologous iliac crest bone graft. Functional outcome was assessed using the age- and sex-specific relative Constant Score.

**Results:**

Mean follow-up was 27 months (range 12–44 months). The relative Constant Score significantly improved from preoperative 61 ± 8 (43–72) to 82 ± 10 (65–100) points at the final follow-up examination (*p* < 0.05). All patients showed bony union radiographically. One patient presented with a re-fracture of the clavicle nearly 3 years after revision surgery and 5 weeks after implant removal. Secondary fractures at the donor site of the anterior superior iliac spine were recorded in two patients.

**Conclusions:**

Iliac crest bone graft and anatomic locking plate fixation allow for a safe and adequate stabilization and radiographical bony union in non- and malunions of the clavicle with a high degree of patient satisfaction. However, secondary fractures of the anterior superior iliac spine constitute relevant complications and the time of hardware removal should be considered carefully.

## Background

Mal- and/or non-union of the clavicle result in several disabling problems for both patient and treating surgeon. Potential symptoms include pain, loss of full range of motion, cosmetic deformity, poor quality of sleep and loss of power. The clavicle is predisposed to non-union due to its subcutaneous position and the influence of fracture associated soft-tissue damage [1]. Incidence of non-unions resulting from midshaft fractures of the clavicle varies between 10.8% following conservative and 3.0% following surgical treatment [2]. High-energy trauma, complete displaced fractures without residual cortical contact between the bony fragments, increased patient age and consumption of tobacco and alcohol seem to be risk factors for potential non-union [3]. The midshaft region is exposed to strong moments of tension and bending as well as torsional forces. Therefore open reduction and internal fixation of midshaft fractures with insufficiently stable implants is at high risk for developing non-unions [4]. Malunion following conservative treatment of clavicle fracture can also result in persistent pain and loss of normal range of shoulder motion [5]. Surgical approaches for treating clavicle fractures include the use of reconstruction plates, tension band wires, dynamic compression plates, elastic-stable intramedullary nailing, Bosworth screws, Knowles pin, semitubular plates and k-wire fixation as well as anatomic preformed plates according to Meves [6–8]. However, in this context the current literature describes numerous complications due to metal implants, but also soft tissue problems and failure of union after surgical treatment for clavicular non-union [9, 10]. Implants providing only insufficient biomechanical stability as well as leading to reduced local blood perfusion and/or bony defects constitute the main reasons for failure of surgical treatment. Therefore the use of anatomic locking compression plates already known for their good results in acute fracture treatment [11] along with additional transplantation of an autologous bone graft depending on the local defect size might present a promising surgical strategy in treatment of clavicular non- and malunions.

## Methods

### Patients and follow-up

All consecutive patients who were treated for clavicular non- or malunion with a locking compression plate and an iliac crest bone graft between January 2010 and December 2014 were included in this retrospective study. The study protocol was approved by the local ethics committee (Ethics Committee of the medical faculty, Klinikum rechts der Isar, Technical University of Munich, Germany; study number 71/15 S).

The preoperative symptoms included pain, limited range of motion, pain when sleeping on the affected side, skin irritation caused by dislocated screws, feeling of weakness of the arm and crepitation.

Standard radiographs of the affected clavicle (anterior-posterior path of rays perpendicular to radiographic cassette, anterior-posterior path of rays 30° tilted cephalad) were performed at the time of the initial presentation of the patient as well as during the routine follow-up examinations in our outpatient clinic. Preoperative planning included the performance of a magnetic resonance imaging (MRI) scan to estimate the non-vital parts of bone to be resected as well as the performance of a computed tomography (CT) scan of both clavicles to measure the original clavicle length to be able to adequately decide if a bone graft would be required. A tricortical iliac crest bone graft was used in all cases with bony defects larger than 15 mm due to the risk of abnormal shoulder biomechanics following clavicular shortening possibly resulting in pain, shoulder motion impairment and loss of strength [12].

The Constant Score was used to assess the shoulder function and activity preoperatively as well as during the routine follow-up examinations in our outpatient clinic. Subsequently the original Constant Score values were converted according to Gerber et al. [13] to receive a normative age- and sex-specific Constant Score (relative Constant Score).

### The implant

The LCP (locking compression plate) superior anterior clavicle plate with the possibility of lateral extension (Depuy-Synthes®, 4528 Zuchwil, Switzerland) is an anatomically precontoured fixation system with three to eight medial shaft holes for 3.5 mm locking or 3.5 mm cortex screws and in case of lateral extension six lateral 2.7 mm divergent locking or 2.4 mm cortex screws. This implant was used for treatment of all enrolled patients.

### Surgical technique and rehabilitation

All patients underwent surgery placed in a beachchair position with the affected arm in a mobile position. A longitudinal skin incision was set below the clavicle with subsequent incision through the clavipectoral fascia also in longitudinal direction to allow for a later closure to ensure sufficient soft tissue coverage. After exposure of the non- or malunion a complex multidimensional osteotomy of the clavicle with medial and lateral axial correction up to vital bone was performed [14]. Vital bone was verified by local blood extravasation resulting from drilling the previously osteotomized bone segments. The tricortical iliac crest bone graft was harvested according to the size assessed in the preoperative CT scan, consecutively adapted to the intraoperative measured size of the bony defect. After implantation of the bone graft the LCP was centered onto the clavicular shaft. At least three screws should be placed medially and laterally to the bone graft to ensure sufficient biomechanical stability. Before drilling the screw holes the plate position was controlled by fluoroscopy. If necessary the iliac crest bone graft should additionally be fixed by a suture cerclage (FiberWire®, Arthrex, Karlsfeld, Germany). After a final radiographical examination the wound was closed layer by layer.

Regarding postoperative rehabilitation primarily the affected arm was immobilized in a sling for 6 weeks. Patients started physiotherapy on the first postoperative day following a standard rehabilitation protocol: abduction and flexion were restricted to 30° for the first two weeks, to 60° for the week three and four postoperative and to 90° for week five and six postoperative. Full weight bearing was not allowed before week 12 postoperative. Contact sports were not allowed before 6 months postoperative. 3, 6, 12 and 24 weeks postoperatively radiographs were performed to evaluate bone healing.

### Statistics

Data is given in terms of the arithmetic mean ± standard deviation and the range in brackets. Preoperative and final relative Constant Scores were compared by calculating the Wilcoxon rank-sum test. A *p*-value <0.05 determined significance. Statistics were calculated using commercially available programs (SigmaStat 3.1, SigmaPlot 8.02, Systat Software Inc., Chicago, USA).

## Results

### Demographics

**Fig. 1:**
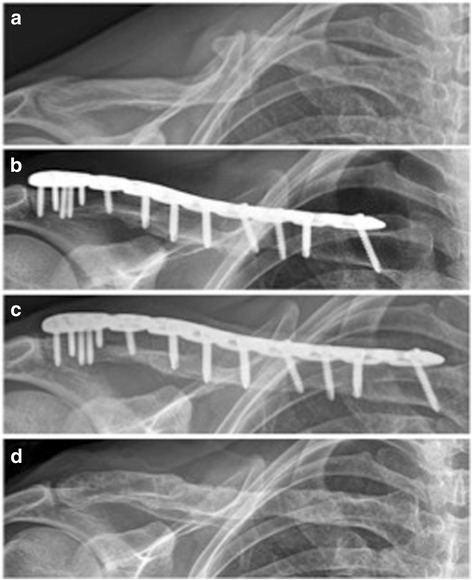
Radiographical outcome of a clavicular non-union (patient No. 10). **a** preoperative; **b** postoperative; **c** 2-year follow-up; **d** after plate removal

Between January 2010 and July 2014, 14 consecutive patients (8 men, 6 women) with a mean age of 44 years (26–67 years) suffering from 11 clavicular non-unions and 3 malunions respectively were enrolled in the study and treated using an iliac crest bone graft and the Synthes® LCP superior anterior clavicle plate (see Table 1). The mean interval between surgery and follow-up was 27 months (range: 12–44 months). The preoperative symptoms included pain (*n* = 12), limited range of motion (*n* = 2), pain when sleeping on the affected side (*n* = 2), skin irritation caused by dislocated screws (*n* = 1), feeling of weakness of the arm (*n* = 1) and crepitation (*n* = 1). In 13 patients the non- or malunion occurred in the midshaft of the clavicle whereas in only one patient the non-union was allocated at the lateral third of the clavicle. In this context Fig. 1 shows the healing process of a patient suffering from a clavicular non-union following non-surgical treatment of a displaced midshaft fracture.

**Table 1:** Patient demographics and outcomes

No.	Age	Sex	Localization	Plate removed	Follow up (months)	p rel CS	f rel CS	Initial fracture treatment	Initial type of implant	Complications
1	39	f	mid	no	37	43	71	s	nail	–
2	67	f	mid	no	44	57	68	n-s	–	–
3	59	m	mid	no	26	57	79	s	plate	–
4	33	f	mid	yes	27	63	95	n-s	–	fracture of the anterior iliac spine
5	54	f	mid	no	12	66	75	n-s	–	–
6	28	m	mid	no	24	50	83	s	plate	–
7	47	m	mid	no	25	69	81	n-s	–	–
8	26	m	mid	yes	23	72	100	n-s	–	fracture of the anterior iliac spine
9	46	f	mid	yes	33	71	95	s	plate	refracture after plate removal
10	46	m	mid	yes	24	62	88	n-s	–	–
11	59	m	lat	no	12	58	65	s	plate	–
12	34	m	mid	no	13	70	84	s	nail	–
13	45	f	mid	yes	35	55	82	n-s	–	–
14	35	m	mid	no	37	62	88	s	plate	–

*No* Number, *p rel CS* preoperative relative Constant Score, *f rel CS* final relative Constant Score, *f* female, *m* male, *mid* midshaft, *lat* lateral, *n-s* non-surgical, *s* surgical

### Surgery characteristics

All three surgeons (CK, PB, SHW) who performed the surgical procedure are experienced upper extremity surgeons. CK performed surgery in 8, PB in 4 and SHW in 2 cases. Due to a defect size larger than 1.5 cm an iliac crest bone graft was used in all patients. Average duration of surgery accounted for 146 min (97–201 min) and the mean dose area product was 46 cGycm^2^ (1, 57–170, 03 cGycm^2^).

### Complications

**Fig. 2:**
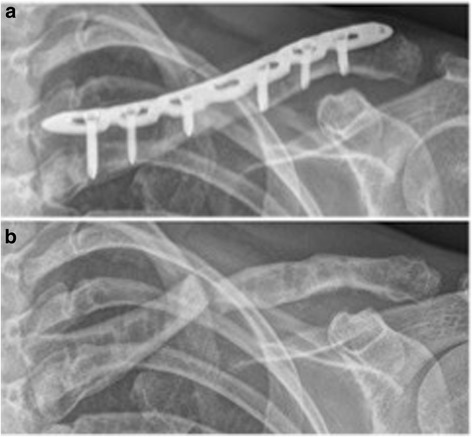
Refracture 5 weeks after implant removal (patient No. 9). **a** before implant removal; **b** 5 weeks after implant removal

In two patients an avulsion of the anterior iliac spine occurred on the 7^th^ (patient No. VIII) and 15^th^ (patient No. IV) postoperative day respectively. In 5 of 14 patients the implant was removed on average 28 months (range 23–35 months) after surgery. Main reasons for hardware removal were the patients’ explicit request due to implant-associated skin irritation while carrying a heavy backpack or sensitivity to weather changes. Patient No. IX presented five weeks after implant removal as well as 33 months postoperatively, with a sudden onset of pain in her left shoulder during swimming. Radiographic control showed a re-fracture of the clavicle (see Fig. 2). There were no further major complications such as wound-healing problems, infection or implant failure to be reported.

### Functional and radiographical outcomes

**Fig. 3:**
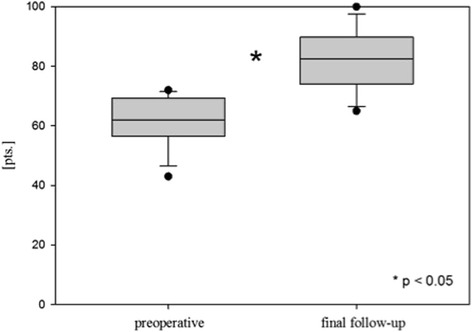
Functional outcome. Preoperative relative Constant Score (preoperative); final relative Constant Score (final follow-up); points (pts.)

The mean age- and sex specific Constant Score significantly improved from preoperative 61 ± 8 points (43–72) to 82 ± 10 points (65–100) after a mean follow-up of 27 months (12–44 months) (*p* < 0.05) (Table 1, Fig. 3). None of the patients reported pain at the final follow-up examination during activities of daily living. All patients were able to return to their regular work. Only one patient complained of mild discomfort during overhead sporting activities (e.g. throwing, tennis). Radiographically bony union occurred in all patients after a mean interval of 8–18 weeks in conventional radiography.

## Discussion

In the present study we report the clinical and radiological mid-term outcome following treatment of clavicular non- and malunions using iliac crest bone grafting and locking plate fixation. The relative Constant Score significantly improved from preoperative 61 ± 8 to 82 ± 10 points at a mean follow-up of 27 months (*p* < 0.05). However two secondary fractures of the anterior superior iliac spine and one re-fracture after implant removal occurred in the presented patient collective.

### Demographics

**Fig. 4:**
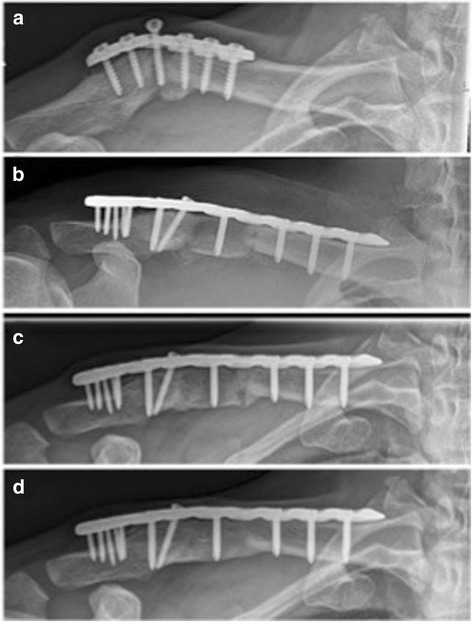
Radiological outcome of a non-union after non-locking plate fixation with only two screws lateral to the fracture gap (patient No. 6). **a** preoperative; **b** postoperative; **c** 1-year follow-up; **d** 2-year follow-up

The presented study collective consists of 14 consecutive patients with a mean age of 41 years and a male–female ratio of 4:3 comparable to other outcome studies concerning age and gender [7, 15, 16]. 9 patients developed non- or malunion despite of previous operation of their acute clavicle fracture in external hospitals. Failure of the initial surgical treatment was most commonly present in case of a biomechanically insufficient fixation as shown in Fig. 4: Patient No. VI was initially treated by a non-locking plate fixation with only two screws in the lateral fragment and one screw in the fracture gap. Therefore the strong moments of tensions and bending as well as torsional forces impacting on the clavicular midshaft led to failure of the plate fixation with secondary dislocation and migration of one screw. The findings of Gilde et al. [17] that non-locking plates result in a higher non-union rate in comparison to locking plates in the treatment of midshaft clavicular fractures confirm the relevance of angle stable implants. In addition locking plates lead to promising results even in high demanding clavicular non-unions such as long standing non-unions with poor bone stock [18] and non-unions of the distal clavicle [19].

### Complications

Major complications in the treatment of clavicular non- and malunion as performed in the presented study contain loss of plate fixation [20], persistent non-union and soft-tissue complications [5]. In the presented study none of these complications were observed. However a total complication rate of 21% is rather high. Anterior iliac crest bone grafting is a very common surgical procedure in patients suffering from non-unions, delayed fracture union or traumatic bone loss [21] to obtain a high qualitative and quantitative bone graft. Common complications contain pain, irritation of the nerve and infection at the donor site. Secondary fractures of the anterior iliac spine, as observed in the current patient collective, are well-known but very rare and only case reports are described in the literature [22]. These patients present with circumscribed pain and loss of range of motion most commonly following an overload of the inserting muscles. In case of non-displaced fractures conservative treatment can lead to bony union without functional residuals as described for patient No. IV. However, in a few cases presenting with severe displacement such as patient No. VIII plate fixation of the anterior iliac spine is indicated and was performed so as described previously. To avoid secondary fractures a sufficient safety distance of at least 2 cm should be maintained in anterior iliac bone graft harvesting [23].

In 5 of 14 enrolled patients elective implant removal was performed by the patients’ explicit request because of complaints such as pain if carrying a heavy backpack or sensitivity to weather changes. In the current literature, routine removal of metal implants remains a controversial issue with a lack of evident guidelines in the surgical treatment of acute clavicle fractures as well as of clavicle non- or malunions. Due to the typical surgical risks and complications, implant removal should only be performed in symptomatic patients after a detailed discussion of potentially available treatment options. Compared to other bony sites of the upper limb, removal of metal implants is most commonly performed in the clavicle [24], most likely because of the prominent subcutaneous position of the implant especially in athletes with poor soft tissue coverage. In our collective, in 5 patients removal of implants was performed at the earliest time point two years after surgery and only after integration of the bone graft and bony union had been proven by CT. However, one patient presented five weeks after elective implant removal with a re-fracture of the clavicle. She reported a sudden onset of pain in her shoulder during swimming. Although swimming constitutes a questionable adequate trauma we recommend to restrain from sport activities for at least 8 weeks after implant removal. However, evidence for a reasonable recommendation is lacking and benefit as well as risks of implant removal have to be investigated in further studies.

### Functional and radiographical outcomes

Follow-up evaluation was performed during the routine follow-up examinations at the outpatient clinic by assessing the Constant Score. The original Constant Score values were converted according to Gerber et al. [13] to receive a normative age- and sex-specific relative Constant Score. At a mean follow-up of 27 months the mean relative Constant Score significantly improved by 21 points in the presented patient collective (*p* < 0.05; see Table 1). The functional results are comparable to other authors who operatively treated non- or malunions of the clavicle. Jubel et al. [7] reported a relative Constant Score of about 95% at a follow-up of 18 months after intramedullary nailing of clavicular non-unions. The authors state that performing surgery without using an iliac crest bone graft constitutes a relevant advantage of intramedullary nailing. However, the requirement of a bone graft is not depending on the used implant but rather on the local bone defect size. In addition intramedullary nailing leads to inferior biomechanical rotational stability in comparison to plate fixation [25]. McKee et al. [20] published the results of non-locking plate fixation in 15 patients suffering from malunions of the clavicle after initial conservative treatment. The authors found a significant improvement of pre- to postoperative self-assessed DASH scores. Nevertheless, in their study loss of plate fixation was seen in one patient and another patient developed a non-union. Hence we consider intramedullary nailing or single non-locking plate fixation in non- or malunions of the clavicle with a significant clavicle shortening in comparison to the healthy contralateral side as an insufficient approach due to the remaining instability allowing for movement with an increased risk of loss of fixation or persisting non-union. In the presented patient collective radiographically bony union occurred in all patients after a mean interval of 8–18 weeks in conventional radiography. In case of implant removal a preoperative computed tomography was performed. However one patient presented five weeks after elective implant removal with a re-fracture of the clavicle. Therefore computed tomography cannot guarantee bony consolidation even in asymptomatic patients.

### Limitations

The major drawbacks of the presented study are its retrospective nature without the assessment of a control group and the low number of included patients. However the most studies focusing on the outcome of clavicular non- or malunions contain a comparable number of cases due to the general low incidence of non-unions of the clavicle. The low incidence is also the reason that we found no comparative study published in the literature. Another drawback presents the postoperative rehabilitation that was performed on an outpatient basis. Thus it was not performed in a standardized way and although physiotherapy should be done according to a standard protocol we cannot guarantee the patient’s compliance.

## Conclusions

Iliac crest bone graft along with anatomic locking plate fixation allow for a sufficient biomechanical stabilization and lead to radiographical bony union in non- and malunions of the clavicle with a high degree of patient satisfaction. However, complications at the donor site such as secondary fractures of the anterior superior iliac spine constitute relevant complications. Hardware removal should be individually discussed and the time of removal should be considered carefully.

## Abbreviations

CT: Computed tomography
DASH: Disability of the arm, shoulder and hand
LCP: Locking compression plate
MRI: Magnetic resonance imaging
MSQ: Munich Shoulder Questionnaire
No: Number
SPADI: Shoulder Pain and Disability Index
